# Supplementary material for: A hidden vulnerable population: Young children up-to-date on vaccine series recommendations except influenza vaccines
Source: PLoS One. 2020 Jun 18;15(6):e0234466. doi: 10.1371/journal.pone.0234466 (PMC7302445; doi:10.1371/journal.pone.0234466)
Supplement: S1 Table — (DOCX) [file pone.0234466.s001.docx]

**Supplementary Table 1 (unabridged version of Table 3 with all comparator outcomes and all covariates): “Change in predicted probabilities of up-to-date vaccine status, multivariate linear probability model regression, U.S children aged 6-23 months old (N=7,246), 2011 NIS.”**

| **Up-to-date status (combinations of seasonal influenza and the 4:3:1:3:3:1:4 series)** | | | | | | |
| --- | --- | --- | --- | --- | --- | --- |
|  | **“BOTH”**  **Both flu and 4:3:1:3:3:1:4 series** | | **“SERIES BUT NOT FLU”**  **4:3:1:3:3:1:4 series, not flu** | | **“NEITHER”**  **Neither flu, 4:3:1:3:3:1:4 series** | |
|  | **ΔPr.** | **95% CI** | **ΔPr.** | **95% CI** | **ΔPr.** | **95% CI** |
| **Child’s race/ethnicity** (ref: non-Hispanic White) |  |  |  |  |  |  |
| Non-Hispanic Black | -0.041 | -0.174, 0.092 | -0.040 | -0.145, 0.092 | 0.038 | -0.090, 0.165 |
| Non-Hispanic other or multiple race | 0.007 | -0.107, 0.121 | 0.001 | -0.018, 0.105 | -0.015 | -0.128, 0.097 |
| Hispanic | 0.044 | -0.070, 0.158 | -0.027 | -0.155, 0.157 | -0.017 | -0.120, 0.086 |
| **Mother is a college graduate** (ref: education less than a college graduate) | ***0.116 | 0.062, 0.170 | *-0.083 | -0.150, -0.016 | *-0.059 | -.0115, -0.002 |
| **Mother never married, widowed, divorced, separated, or deceased** (ref: married) | 0.004 | -0.064, 0.071 | 0.009 | -0.090, 0.108 | -0.007 | -0.102, 0.088 |
|  |  |  |  |  |  |  |
| **Child’s race/ethnicity*mother’s education** |  |  |  |  |  |  |
| (Ref: non-Hispanic White with college graduate mother) |  |  |  |  |  |  |
| Non-Hispanic Black with college graduate mother | -0.161 | -0.325, 0.003 | 0.121 | -0.094, 0.336 | 0.130 | -0.097, 0.357 |
| Non-Hispanic other/multiple race with college graduate mother | -0.050 | -0.195, 0.095 | 0.058 | -0.122, 0.238 | 0.022 | -0.111, 0.155 |
| Hispanic with college graduate mother | **-0.208 | -0.350, -0.065 | **0.263 | 0.104, 0.422 | -0.032 | -0.153, 0.088 |
| **Child’s race/ethnicity*mother’s marital status** |  |  |  |  |  |  |
| (Ref: non-Hispanic White; mother never married, widowed, divorced, separated, or deceased) |  |  |  |  |  |  |
| Non-Hispanic Black; mother never married, widowed, divorced, separated, or deceased | -0.044 | -0.201, 0.113 | 0.022 | -0.157, 0.202 | 0.030 | -0.128, 0.187 |
| Non-Hispanic other/multiple race; mother never married, widowed, divorced, separated, or deceased | 0.063 | -0.107, 0.234 | -0.042 | -0.253, 0.169 | -0.023 | -0.190, 0.143 |
| Hispanic; mother never married, widowed, divorced, separated, or deceased | -0.080 | -0.211, 0.051 | 0.068 | -0.082, 0.217 | 0.004 | -0.124, 0.133 |
| **Mom is college graduate*never married/widowed/divorced/separated/deceased** (ref: mom is college graduate*married) | 0.011 | -0.156, 0.177 | -0.080 | -0.240, 0.081 | 0.114 | -0.074, 0.302 |
|  |  |  |  |  |  |  |
| **Child’s race/ethnicity*mother’s education*mother’s marital status** |  |  |  |  |  |  |
| (Ref: non-Hispanic White; mom is college graduate; never married, widowed, divorced, separated, or deceased) |  |  |  |  |  |  |
| Non-Hispanic Black; mom is college graduate; never married, widowed, divorced, separated, or deceased | 0.119 | -0.160, 0.398 | 0.086 | -0.258, 0.430 | -0.253 | -0.588, 0.083 |
| Non-Hispanic other/multiple race; mom is college graduate; never married, widowed, divorced, separated, or deceased | 0.141 | -0.368, 0.649 | 0.076 | -0.357, 0.510 | -0.202 | -0.498, 0.094 |
| Hispanic; mom is college graduate; never married, widowed, divorced, separated, or deceased | -0.046 | -0.290, 0.197 | -0.115 | -0.475, 0.244 | 0.140 | -0.232, 0.512 |
|  |  |  |  |  |  |  |
| **Covariates** |  |  |  |  |  |  |
| Child is male | 0.004 | -0.034, 0.043 | 0.010 | -0.034, 0.053 | -0.013 | -0.050, 0.024 |
| Child is first born | 0.036 | -0.001, 0.073 | 0.041 | -0.006, 0.087 | ***-0.082 | -0.122, -0.041 |
| Child ever received WIC benefits | -0.028 | -0.081, 0.026 | 0.025 | -0.045, 0.095 | 0.002 | -0.066, 0.070 |
| Child uninsured | -0.013 | -0.077, 0.050 | -0.032 | -0.119, 0.056 | 0.054 | -0.021, 0.130 |
| Mother’s age group (ref: ≥30 years) |  |  |  |  |  |  |
| ≤19 years | -0.057 | -0.174, 0.060 | 0.078 | -0.083, 0.239 | 0.032 | -0.104, 0.169 |
| 20-29 years | *0.051 | -0.100, -0.002 | -0.006 | -0.060, 0.047 | *0.050 | 0.007, 0.093 |
| English language (vs. Spanish or other) | -0.021 | -0.119, 0.078 | 0.061 | -0.035, 0.157 | -0.037 | -0.115, 0.041 |
| Housing arrangement (ref: owned or being bought) |  |  |  |  |  |  |
| Rented | 0.014 | -0.032, 0.060 | -0.050 | -0.103, 0.003 | 0.041 | -0.007, 0.088 |
| Other arrangement | *-0.090 | -0.164, -0.016 | 0.010 | -0.111, 0.130 | 0.082 | -0.019, 0.183 |
| Provider facility type (ref: Private) |  |  |  |  |  |  |
| Public/WIC | *-0.068 | -0.134, -0.002 | 0.017 | -0.061, 0.095 | *0.081 | 0.014, 0.148 |
| Hospital | -0.051 | -0.108, 0.005 | -0.009 | -0.085, 0.067 | 0.016 | -0.043, 0.075 |
| Military/other facilities | *-0.113 | -0.196, -0.030 | -0.032 | -0.155, 0.091 | **0.174 | 0.054, 0.293 |
| Mixed | 0.017 | -0.042, 0.076 | 0.036 | -0.032, 0.104 | -0.048 | -0.099, 0.003 |
| Child was never breastfed nor fed breast milk (ref: ever) | *-0.056 | -0.100, -0.015 | 0.033 | -0.023, 0.088 | 0.018 | -0.030, 0.066 |
| Parent ever refused/decided not to have their child vaccinated (ref: never) | ***-0.130 | -0.173, -0.087 | **0.099 | 0.042, 0.157 | *0.052 | 0.002, 0.101 |
| Parent ever delayed or put off having their child vaccinated (ref: never) | ***-0.083 | -0.124, -0.042 | **-0.075 | -0.125, -0.026 | ***0.155 | 0.108, 0.202 |
| Parent belief that vaccines are necessary to protect children’s health | *0.015 | 0.002, 0.028 | 0.008 | -0.011, 0.026 | **-0.022 | -0.038, -0.006 |
| Parent belief that vaccines do a good job at preventing their diseases | -0.009 | -0.026, 0.008 | 0.012 | -0.005, 0.030 | -0.007 | -0.023, 0.009 |
| Parent belief that vaccines are safe | *0.014 | 0.003, 0.024 | 0.000 | -0.012, 0.013 | *-0.011 | -0.022, -0.001 |
| Parent belief that vaccine-preventable diseases are serious, can hurt children | -0.000 | -0.010, 0.001 | -0.006 | -0.018, 0.007 | 0.004 | -0.006, 0.015 |
| Parent perception of strength of physician vaccine recommendation | -0.004 | -0.022, 0.015 | 0.002 | -0.014, 0.019 | 0.005 | -0.006, 0.017 |

Source: 2011 National Immunization Survey (NIS) data, children represented in the Parental Concerns module with provider-verified vaccination data and eligible for the influenza vaccination up-to-date question who are not missing any covariates. “ΔPr.” represents changes in predicted probabilities, weighted to be nationally-representative (e.g., “0.116” in the means an absolute increase in probability of the up-to-date status outcome associated with change in the covariate; this is the same as an 11.6 percentage point absolute increase in chance of up-to-date status outcome associated with change in the covariate). Standard errors used to calculate 95% confidence intervals are adjusted for complex survey design. Also controls for geographical area of residence (not shown due to large number of variable values). For the last 5 covariates (parent beliefs/perceptions), the scale is 0-10 where 0 is disagree and 10 is agree. For the “series but not flu” outcome, shaded cells represent significant coefficients indicating vulnerability unique to the “series not flu” outcome or in a direction different than suggested from the “both” or “neither” outcomes. *p<0.05 **p<0.01 ***p<0.001
