# Supplementary material for: A hidden vulnerable population: Young children up-to-date on vaccine series recommendations except influenza vaccines
Source: PLoS One. 2020 Jun 18;15(6):e0234466. doi: 10.1371/journal.pone.0234466 (PMC7302445; doi:10.1371/journal.pone.0234466)
Supplement: S2 Table — (DOCX) [file pone.0234466.s002.docx]

**Supplementary Table 2 (unabridged version of Table 4 with all comparator outcomes): “Predicted probabilities of up-to-date vaccine outcomes among intersectional interaction term subgroups, multivariate linear probability model regression, U.S children aged 6-23 months old (N=7,246), 2011 NIS.”**

| **Up-to-date status (combinations of seasonal influenza and the 4:3:1:3:3:1:4 series)** | | | | | | |
| --- | --- | --- | --- | --- | --- | --- |
|  | **“ BOTH”**  **Both flu and 4:3:1:3:3:1:4 series** | | **“SERIES BUT NOT FLU”**  **4:3:1:3:3:1:4 series, not flu** | | **“NEITHER”**  **Neither flu, 4:3:1:3:3:1:4 series** | |
| ***Main coefficient subgroups*** | **Pr.** | **95% CI** | **Pr.** | **95% CI** | **Pr.** | **95% CI** |
| **Child’s race/ethnicity** |  |  |  |  |  |  |
| Non-Hispanic White only | 0.295 | 0.270, 0.321 | 0.419 | 0.386, 0.453 | 0.230 | 0.200, 0.260 |
| Non-Hispanic Black only | 0.186 | 0.126, 0.246 | 0.434 | 0.358, 0.509 | 0.315 | 0.236, 0.395 |
| Non-Hispanic other or multiple race | 0.309 | 0.251, 0.368 | 0.430 | 0.362, 0.498 | 0.208 | 0.158, 0.258 |
| Hispanic | 0.237 | 0.186, 0.288 | 0.506 | 0.448, 0.564 | 0.207 | 0.163, 0.251 |
| **Mother’s education** |  |  |  |  |  |  |
| Less than a college graduate | 0.250 | 0.222, 0.278 | 0.448 | 0.414, 0.482 | 0.248 | 0.220, 0.276 |
| College graduate | 0.296 | 0.259, 0.332 | 0.429 | 0.378, 0.479 | 0.225 | 0.178, 0.272 |
| **Mother’s marital status** |  |  |  |  |  |  |
| Married | 0.284 | 0.254, 0.314 | 0.427 | 0.395, 0.460 | 0.229 | 0.200, 0.258 |
| Never married, widowed, divorced, separated, or deceased | 0.276 | 0.229, 0.324 | 0.424 | 0.374, 0.474 | 0.259 | 0.208, 0.309 |
| ***Two-way interaction term subgroups*** |  |  |  |  |  |  |
| **Child’s race/ethnicity*mother’s education** |  |  |  |  |  |  |
| Non-Hispanic White child; non-college graduate mother | 0.253 | 0.220, 0.286 | 0.452 | 0.407, 0.498 | 0.247 | 0.205, 0.289 |
| Non-Hispanic White child; college graduate mother | 0.372 | 0.316, 0.429 | 0.344 | 0.291, 0.396 | 0.225 | 0.163, 0.287 |
| Non-Hispanic Black child; non-college graduate mother | 0.198 | 0.106, 0.290 | 0.419 | 0.323, 0.516 | 0.294 | 0.212, 0.377 |
| Non-Hispanic Black child; college graduate mother | 0.194 | 0.115, 0.274 | 0.460 | 0.331, 0.589 | 0.321 | 0.180, 0.462 |
| Non-Hispanic other or multiple race child; non-college graduate mother | 0.280 | 0.199, 0.362 | 0.439 | 0.335, 0.544 | 0.224 | 0.152, 0.296 |
| Non-Hispanic other or multiple race child; college graduate mother | 0.395 | 0.240, 0.550 | 0.413 | 0.284, 0.543 | 0.159 | 0.087, 0.232 |
| Hispanic child; non-college graduate mother | 0.272 | 0.193, 0.350 | 0.447 | 0.370, 0.524 | 0.232 | 0.170, 0.293 |
| Hispanic child; college graduate mother | 0.168 | 0.100, 0.236 | 0.565 | 0.447, 0.683 | 0.222 | 0.116, 0.329 |
| **Child’s race/ethnicity*mother’s marital status** |  |  |  |  |  |  |
| Non-Hispanic White child; married mother | 0.294 | 0.264, 0.324 | 0.419 | 0.375, 0.464 | 0.228 | 0.183, 0.273 |
| Non-Hispanic White child; never married, widowed, divorced, separated, or deceased mother | 0.301 | 0.236, 0.367 | 0.399 | 0.332, 0.466 | 0.262 | 0.190, 0.335 |
| Non-Hispanic Black child; married mother | 0.194 | 0.105, 0.284 | 0.423 | 0.320, 0.526 | 0.313 | 0.211, 0.415 |
| Non-Hispanic Black child; never married, widowed, divorced, separated, or deceased mother | 0.201 | 0.131, 0.271 | 0.457 | 0.360, 0.553 | 0.285 | 0.210, 0.360 |
| Non-Hispanic other or multiple race child; married mother | 0.283 | 0.210, 0.356 | 0.441 | 0.346, 0.536 | 0.221 | 0.156, 0.285 |
| Non-Hispanic other or multiple race child; never married, widowed, divorced, separated, or deceased mother | 0.404 | 0.227, 0.582 | 0.407 | 0.258, 0.555 | 0.158 | 0.069, 0.248 |
| Hispanic child; married mother | 0.263 | 0.190, 0.336 | 0.488 | 0.415, 0.561 | 0.200 | 0.144, 0.255 |
| Hispanic child; never married, widowed, divorced, separated, or deceased mother | 0.173 | 0.113, 0.234 | 0.494 | 0.377, 0.611 | 0.289 | 0.172, 0.406 |
| **Mother’s education*mother’s marital status** |  |  |  |  |  |  |
| Mother is not a college graduate; married | 0.260 | 0.220, 0.299 | 0.437 | 0.393, 0.481 | 0.248 | 0.209, 0.287 |
| Mother is not a college graduate; never married, widowed, divorced, separated, or deceased | 0.241 | 0.202, 0.281 | 0.463 | 0.411, 0.516 | 0.244 | 0.199, 0.288 |
| Mother is a college graduate; married | 0.294 | 0.258, 0.330 | 0.447 | 0.396, 0.498 | 0.199 | 0.157, 0.240 |
| Mother is a college graduate; never married, widowed, divorced, separated, or deceased | 0.301 | 0.206, 0.396 | 0.380 | 0.270, 0.490 | 0.296 | 0.176, 0.417 |
| ***Three-way interaction term subgroups*** |  |  |  |  |  |  |
| **Child’s race/ethnicity*mother’s education*mother’s marital status** |  |  |  |  |  |  |
| Non-Hisp. White child; mother is not college grad; married | 0.252 | 0.211, 0.292 | 0.449 | 0.391, 0.508 | 0.249 | 0.190, 0.308 |
| Non-Hisp. White child; mother is not college grad; never married/widowed/divorced/separated/deceased | 0.256 | 0.200, 0.311 | 0.458 | 0.382, 0.534 | 0.242 | 0.178, 0.306 |
| Non-Hisp. White child; mother is college grad; married | 0.368 | 0.329, 0.406 | 0.366 | 0.319, 0.413 | 0.191 | 0.153, 0.228 |
| Non-Hisp. White child; mother is college grad; never married/widowed/divorced/separated/deceased | 0.382 | 0.231, 0.533 | 0.295 | 0.166, 0.424 | 0.297 | 0.129, 0.466 |
| Non-Hisp. Black child; mother is not college grad; married | 0.211 | 0.081, 0.341 | 0.409 | 0.276, 0.543 | 0.287 | 0.172, 0.402 |
| Non-Hisp. Black child; mother is not college grad; never married/widowed/divorced/separated/deceased | 0.170 | 0.102, 0.239 | 0.441 | 0.351, 0.530 | 0.309 | 0.234, 0.385 |
| Non-Hisp. Black child; mother is college grad; married | 0.165 | 0.072, 0.259 | 0.448 | 0.286, 0.609 | 0.358 | 0.166, 0.550 |
| Non-Hisp. Black child; mother is college grad; never married/widowed/divorced/separated/deceased | 0.255 | 0.108, 0.401 | 0.485 | 0.275, 0.696 | 0.242 | 0.085, 0.399 |
| Non-Hisp. other/multiple race child; mother is not college grad; married | 0.259 | 0.154, 0.364 | 0.450 | 0.308, 0.592 | 0.234 | 0.141, 0.327 |
| Non-Hisp. other/multiple race child; mother is not college grad; never married/widowed/divorced/separated/deceased | 0.326 | 0.207, 0.444 | 0.417 | 0.292, 0.542 | 0.204 | 0.095, 0.313 |
| Non-Hisp. other/multiple race child; mother is college grad; married | 0.325 | 0.237, 0.413 | 0.425 | 0.333, 0.518 | 0.197 | 0.123, 0.271 |
| Non-Hisp. other/multiple race child; mother is college grad; never married/widowed/divorced/separated/deceased | 0.543 | 0.097, 0.988 | 0.388 | 0.040, 0.737 | 0.079 | 0.000, 0.239 |
| Hispanic child; mother is not college grad; married | 0.296 | 0.191, 0.402 | 0.423 | 0.325, 0.520 | 0.232 | 0.155, 0.310 |
| Hispanic child; mother is not college grad; never married/widowed/divorced/separated/deceased | 0.220 | 0.149, 0.291 | 0.499 | 0.411, 0.588 | 0.230 | 0.158, 0.302 |
| Hispanic child; mother is college grad; married | 0.204 | 0.123, 0.286 | 0.603 | 0.489, 0.717 | 0.142 | 0.067, 0.216 |
| Hispanic child; mother is college grad; never married/widowed/divorced/separated/deceased | 0.092 | 0.000, 0.209 | 0.485 | 0.206, 0.763 | 0.393 | 0.098, 0.687 |

Source: 2011 National Immunization Survey (NIS) data, children represented in the Parental Concerns module with provider-verified vaccination data and eligible for the influenza vaccination up-to-date question who are not missing any covariates from main analysis. Coefficients represent predicted linear probabilities of vaccination up-to-date outcomes among all hierarchical interaction term subgroups from multivariate linear probability regression models (Table 3).
