# Supplementary material for: A hidden vulnerable population: Young children up-to-date on vaccine series recommendations except influenza vaccines
Source: PLoS One. 2020 Jun 18;15(6):e0234466. doi: 10.1371/journal.pone.0234466 (PMC7302445; doi:10.1371/journal.pone.0234466)
Supplement: Attachment — Submitted filename: Response to reviewers 2020 04 13.docx [file pone.0234466.s003.docx]

**Comments from the editor**:

Thank you for submitting your manuscript to PLOS ONE. After careful consideration, we feel that it has merit but does not fully meet PLOS ONE’s publication criteria as it currently stands. Therefore, we invite you to submit a revised version of the manuscript that addresses the points raised during the review process.

While I think this is an important topic that warrants investigation, there were several issues with both the study design, and the manuscript itself that are significant enough that they seriously undermine the contributions of the study. The manuscript has a number of weaknesses which need to be considered.

The PLOS ONE publish research on the basis of scientific validity and rigorous methodology. Together with both reviewers I have a number of reservations about this paper regarding both above mentioned issues. They are outlined below.

According to the journal criteria  an analytical, cross-sectional study, like this one must has been conducted rigorously. However, the methods used are not clearly presented.  The work contains some methodological mistakes which diminish its quality. I suggest to reconsider the methods used (see the list of reviewers’ comments below).

- Thank you for your comments and the opportunity to revise. We have revised the methods to include a short, high-level roadmap up front in the “Analysis” sub-section of the Methods section before getting into the details. We hope this clarifies what descriptive statistics and regressions were run and how findings were interpreted and visualized.
- Similarly, in the presentation of results, following a suggestion from a reviewer we moved the full, large results from Tables 3 and 4 to two supplementary tables. Instead, in the main text we present substantially shortened versions of Tables 3 and 4 that only show key findings for the main outcome variable (noting in the footnotes what outcome variable columns and control variable rows are not shown, and pointing readers to new supplementary tables for full output).
- Additionally, we made numerous other additions and clarifications on Methods related to reviewers as detailed on the following pages, such as linking to official federal government measure definitions for our outcome variables, and adding information on the scale of the parental concerns module variables with citations.
- These changes should allow for the methods to be easily digestible in shorter form and better navigated with an upfront roadmap and also answer reviewers’ methodological questions.

1. Please ensure that your manuscript meets PLOS ONE's style requirements, including those for file naming. The PLOS ONE style templates can be found at <http://www.plosone.org/attachments/PLOSOne_formatting_sample_main_body.pdf> and <http://www.plosone.org/attachments/PLOSOne_formatting_sample_title_authors_affiliations.pdf>

- These hyperlinks above do not work, but our original submission and this resubmission are in compliance with the [PLOS ONE instructions for authors submission guidelines online](https://journals.plos.org/plosone/s/submission-guidelines).

2.  Thank you for stating in your Funding Statement:

"We acknowledge Pennsylvania State University's Department of Health Policy and Administration, Demography program, and Population Research Institute for supporting this research. The Population Research Institute is supported by an infrastructure grant from NIH (2R24HD041025-11).

This publication was also supported, in part, by Grant UL1 TR000127 and KL2 TR000126 from the National Center for Advancing Translational Sciences (NCATS)."

Please provide an amended statement that declares *all* the funding or sources of support (whether external or internal to your organization) received during this study, as detailed online in our guide for authors at <http://journals.plos.org/plosone/s/submit-now>.  Please also include the statement “There was no additional external funding received for this study.” in your updated Funding Statement.

Please include your amended Funding Statement within your cover letter. We will change the online submission form on your behalf.

- We moved some support statements out of the Acknowledgements section and to a new Funding Statement section. We copied this into the cover letter as well. It reads:

  “**Funding Statement**

  There was no external funding received for this study. We acknowledge indirect support provided by Pennsylvania State University's Department of Health Policy and Administration, Demography program, and Population Research Institute. The Population Research Institute is supported by an infrastructure grant from NIH (2R24HD041025-11). This publication was also supported, in part, by Grant UL1 TR000127 and KL2 TR000126 from the National Center for Advancing Translational Sciences (NCATS). The funders provided support in the form of salaries or infrastructure for authors WKB and PYM, but did not have any additional role in the study design, data collection and analysis, decision to publish, or preparation of the manuscript. The specific roles of these authors are articulated in the ‘author contributions’ section.”

3. Thank you for stating the following in the Competing Interests section:

"William K. Bleser discloses consulting fees from Merck unrelated to this research. Daniel A. Salmon discloses consulting fees and research grants from Merck, Pfizer, and Walgreens unrelated to this research. No other financial disclosures were reported."

We note that one or more of the authors are employed by a commercial company: Merck, Pfizer, and Walgreens

- You’re welcome. Note that these are not our full-time employers, these were one-time consultancies and were not related to this research.

4.     Please provide an amended Funding Statement declaring this commercial affiliation, as well as a statement regarding the Role of Funders in your study. If the funding organization did not play a role in the study design, data collection and analysis, decision to publish, or preparation of the manuscript and only provided financial support in the form of authors' salaries and/or research materials, please review your statements relating to the author contributions, and ensure you have specifically and accurately indicated the role(s) that these authors had in your study. You can update author roles in the Author Contributions section of the online submission form.

Please also include the following statement within your amended Funding Statement.

“The funder provided support in the form of salaries for authors [insert relevant initials], but did not have any additional role in the study design, data collection and analysis, decision to publish, or preparation of the manuscript. The specific roles of these authors are articulated in the ‘author contributions’ section.”

If your commercial affiliation did play a role in your study, please state and explain this role within your updated Funding Statement.

- The competing interests statement organizations below were totally unrelated to this research. We added the following to the Funding Statement:

  “The funders provided support in the form of salaries or infrastructure for authors WKB and PYM, but did not have any additional role in the study design, data collection and analysis, decision to publish, or preparation of the manuscript. The specific roles of these authors are articulated in the ‘author contributions’ section.”
- We added the section “Author Contributions” per request and in accordance with [PLOS ONE’s instructions adopting the CRediT Taxonomy](https://journals.plos.org/plosone/s/authorship#loc-author-contributions):

  “**Author Contributions**

  William K. Bleser. Roles: Conceptualization, data curation, formal analysis, funding acquisition, methodology, project administration, visualization, writing – original draft preparation, writing – review & editing.

Daniel A. Salmon. Roles: Methodology, supervision, writing – review & editing.

Patricia Y. Miranda. Roles: Conceptualization, funding acquisition, methodology, project administration, supervision, writing – review & editing.”

5. Please also provide an updated Competing Interests Statement declaring this commercial affiliation along with any other relevant declarations relating to employment, consultancy, patents, products in development, or marketed products, etc.

- We moved some competing interests statements out of the Acknowledgements section and to a new Competing Interests section. We copied this into the cover letter as well. It reads:

  “**Competing Interests**

  William K. Bleser discloses past consulting fees from Merck unrelated to this research. Daniel A. Salmon discloses consulting fees and research grants from Merck, Pfizer, and Walgreens unrelated to this research. No other financial disclosures were reported. This does not alter our adherence to PLOS ONE policies on sharing data and materials.”

Within your Competing Interests Statement, please confirm that this commercial affiliation does not alter your adherence to all PLOS ONE policies on sharing data and materials by including the following statement: "This does not alter our adherence to  PLOS ONE policies on sharing data and materials.” (as detailed online in our guide for authors <http://journals.plos.org/plosone/s/competing-interests>) . If this adherence statement is not accurate and  there are restrictions on sharing of data and/or materials, please state these. Please note that we cannot proceed with consideration of your article until this information has been declared.

- We added the statement as requested above to our Competing Interests section.

Please include both an updated Funding Statement and Competing Interests Statement in your cover letter. We will change the online submission form on your behalf.

- We added the updated Funding Statement and Competing Interests sections to the cover letter.

Please know it is PLOS ONE policy for corresponding authors to declare, on behalf of all authors, all potential competing interests for the purposes of transparency. PLOS defines a competing interest as anything that interferes with, or could reasonably be perceived as interfering with, the full and objective presentation, peer review, editorial decision-making, or publication of research or non-research articles submitted to one of the journals. Competing interests can be financial or non-financial, professional, or personal. Competing interests can arise in relationship to an organization or another person. Please follow this link to our website for more details on competing interests: <http://journals.plos.org/plosone/s/competing-interests>

- These are noted in the Competing Interests section. None of these were related to or affected the research but we have declared them for transparency.

6. We noted in your submission details that a portion of your manuscript may have been presented or published elsewhere. ["An earlier version of this manuscript was published as a part of a dissertation. "] Please clarify whether this [conference proceeding or publication] was peer-reviewed and formally published. If this work was previously peer-reviewed and published, in the cover letter please provide the reason that this work does not constitute dual publication and should be included in the current manuscript.

- It was not published as part of any conference proceedings or peer-reviewed publication. However, an older version of this manuscript was part of William K. Bleser’s doctoral dissertation and was posted in the Pennsylvania State University’s “Dissertations & Theses” database as required by Pennsylvania State University. This is not a formal publication; it is a final, accepted dissertation repository.

7. Your ethics statement must appear in the Methods section of your manuscript. If your ethics statement is written in any section besides the Methods, please move it to the Methods section and delete it from any other section. Please also ensure that your ethics statement is included in your manuscript, as the ethics section of your online submission will not be published alongside your manuscript.

- The ethics statement was already contained in the Methods section (first paragraph) so we removed it from the Acknowledgements section.

**Comments from Reviewer #1**:

Reviewer #1: This manuscript presents an analysis of nationally representative survey data on influenza vaccine uptake in young children. The authors investigate cross-sectional associations with a number of predictors of vaccination, including social and demographic variables and past vaccine utilization. Importantly, the analysis also seeks to identify intersectionality, which is a novel and important approach that represents a significant contribution to the literature on vaccine hesitancy. Overall, the analysis is rigorous, the conclusions are supported by the data, and the manuscript is well-written and clear. I agree with the authors' conclusion that it is concerning that there may influenza vaccine specific hesitancy, even among parents who are regularly choosing vaccination for their children to prevent other diseases. I have some questions and comments, which I believe would strengthen the manuscript. These comments are separated by manuscript section below.

- Thank you for the accurate summary and kind words of the importance and quality of the work.
- We have addressed your section-by-section comments below.

Introduction

It is important that the authors are clear about their assertions about risk of influenza and benefits of influenza vaccine. Specifically, children < 5 years old are typically considered to be at lower risk of influenza infection than school aged children (most likely due to to contact patterns), unless they attend child care outside of the home. The references cited here are reporting risk of complications due to influenza infection (e.g. pneumonia) which children <5 ARE considered to be at especially high risk for developing. I suggest a minor change to the language to indicate that these children are at higher risk of complications, rather than saying they are simply at high risk of influenza. Similarly, influenza vaccination is the most effective preventive measure for prevention of infection.

- Thank you for this simple yet important clarification. The first sentence of the introduction was the only place where this point was made, so we amended it to refer to influenza complications risk instead of influenza risk.

Methods:

The scale for parent belief variables should be stated, otherwise the mean and SD values reported in table 1 are difficult to interpret for someone who is not familiar with these data.

- Thanks for pointing out that the scale of the questions were unclear. This is important context for the readers. The scale is already included in the footnotes of tables, but we agree that it should be included in the text. Thus, we have added the following description of the survey instruments for the Parental Concerns module questions in the “Determinants of influence vaccination” sub-section of the Methods section (**new text in bold**, including a new source citing the survey instrument).

**The Parental Concerns module data are restricted and not contained in the public use dataset, but these variables were obtained by the authors and analyzed in a Research Data Center for his study. However, the survey instrument is publicly available online [36]. Questions 1-5 below ask parents to rate the statement on a scale of 0-10 where 0 is “strongly disagree” and 10 is “strongly agree.” Questions 6 and 7 below ask parents if they have ever refused or delayed getting their child vaccinated (binary question):**

1) *vaccines are necessary to protect child health* [26,45–50];
2) *vaccines do a good job at preventing their diseases* [26,45–50];
3) *vaccines are safe* [45–47,51];
4) *vaccine-preventable diseases are serious and can hurt children* [26,46,52];
5) *strength of physician vaccine recommendation* [27,41,45–50,52–56];
6) *history of refusing their child’s vaccines*; and
7) *history of delaying their child’s vaccines*.

   **[New citation]:**
   36. NORC at the University of Chicago. NIS-Child Hard Copy Questionnaire: Q4/2011. 2011. Available: ftp://ftp.cdc.gov/pub/Health_Statistics/NCHS/Dataset_Documentation/NIS/NISPUF11_HHQuex.pdf

The primary outcome of interest is potentially problematic due to the annual nature of influenza vaccines. Unless I am misunderstanding this outcome, a 2 year old child who received two doses of influenza vaccine in the year of the survey (2011) but was unvaccinated in the previous year (2010) would be considered to have "hidden vulnerability" to influenza. This is true, even though, by ACIP recommendations that child would be considered fully vaccinated against influenza for the 2011 season. In addition, that child may actually have higher antibody titres to vaccine strains and be expected to have higher vaccine effectiveness (due to negative interference) for the 2011 season. At a minimum, I recommend a sensitivity analysis where children are classified as "fully vaccinated", "partially vaccinated" or "unvaccinated" against influenza using ACIP guidelines to determine this categorization.

- Thanks for asking for clarification. The influenza vaccination outcome variable is defined by the US federal government to assess complete child influenza vaccination coverage by the child’s second birthday. It is based off of the child’s birthdate, and whether they have had the appropriate number of seasonal flu vaccine by their second birthday across however many flu seasons that two-year period covered and depending on when the survey questions was asked to them (i.e., the number of vaccines required to be considered “up to date” can vary depending on birthdate and survey administration date). Nonetheless, it is vetted by the CDC. The full description is below from the CDC survey manual ([click here and see pages 65-66](ftp://ftp.cdc.gov/pub/health_statistics/nchs/dataset_documentation/nis/nispuf11_dug.pdf), also source 38 in the paper):

“Vaccinated – For interviews conducted during year x (defined using year variable associated with the quarter), child was of age between 6 and 23 months during the entire span from 9/1 through 12/31 of year x-1, and either a) received no doses of influenza vaccine prior to 9/1/x-1, but then received two between 9/1/(x-1) and whichever is earlier, date of interview or 1/31/x or b) received at least one dose of influenza vaccine prior to 9/1/x-1 and then received one during the period 9/1/x-1 through 12/31/x-1.

Not vaccinated – For interviews conducted during year x (defined using year variable associated with the quarter), child was of age between 6 and 23 months during the entire span from 9/1 through 12/31 of year x-1, but does not qualify for the above definition.

Not eligible – For interviews conducted during year x (defined using year variable associated with the quarter), child's age fell outside the span of 6 and 23 months at any point between 9/1/x-1 and 12/31/x-1.”

- For the reader’s aid, we clarified and added the following guided language to the “Dependent variable” sub-section of the Methods section, including where to find more information:

  “The first is complete influenza vaccination – that is, whether the child received the full number of **seasonal** influenza vaccines given the number of influenza seasons they have experienced by their second birthday **and when the survey was administered** (children not 6-23 months of age during the span of September 1 to December 31 are “not eligible;” **see Section 7.8.1 and Table 7 of the survey user’s guide [38]**).”

Was there any correction for multiple testing? Why or why not?

- Thank you for raising this point, which we should clarify. Multiple testing is not warranted here nor are we concerned about multiple comparisons issues given we were not asking the question: “is at least one of these variables significant?” (i.e., we did not claim the study as having overall significant findings by looking for a single p-value across a whole set of p-values). Rather, we are asking “which key variables exhibit statistically significant disparities?” where the variables were *a priori* chosen using a conceptual model (Andersen’s model of health services use).

  Further, we focused on just three x-variables, again chosen *a priori* from the conceptual model and prior literature on intersectionality: child’s race/ethnicity, mother’s education, and mother’s marital status. Other x-variables were included as control variables.

  This approach follows general recommendations to avoid multiple comparisons issues:
  - Feise, R. J. (2002). Do multiple outcome measures require p-value adjustment? BMC Medical Research Methodology, 2, 8. http://doi.org/10.1186/1471-2288-2-8
  - Gelman, A., Hill, J., & Yajima, M. (2012). Why We (Usually) Don’t Have to Worry About Multiple Comparisons. Journal of Research on Educational Effectiveness, 5(2), 189–211. http://doi.org/10.1080/19345747.2011.618213

That said, we made sure in our manuscript language to avoid overinterpreting findings, especially in light of this being a cross-sectional study due to key variables being asked in only one survey year. We took care to make sure we used language referencing “associations” and not causal language, were careful to use revised language such as X “may be” associated with Y, instead of “is,” and pointed toward more research being required.

Results: The tables are difficult to read and interpret due to the large number of variables and interactions tested. I would recommend that the authors present interesting findings and relevant supporting information in the main tables and move everything else to supplemental tables. Another possibility is to present only the "series but not flu" outcome in the main tables and move other outcomes to a supplementary table.

- Thank you for this suggestion. We agree that this would be helpful for brevity and making key results more digestible.

  For Tables 1 and 2 (descriptive statistics of the study population, and bivariate correlates of each covariate with each outcome variable), we leave in the full tables because they are useful for readers to understand the study population and the variation among the outcome.

  For Table 3, we preserved the original whole table as a new Supplementary Table 1 and then in the paper cut down the table in two ways, per suggestion. First, we now only show the “series but not flu” outcome, and (2) we only show the three main x-variables, their interaction terms, and the significant covariates indicating vulnerability unique to the “series not flu” outcome or in a direction different than suggested from the “both” or “neither” outcomes (i.e., the shaded cells). We add to the footnotes the names of the covariates that we controlled for but which are not shown in the table, and refer readers to Supplementary Table 1 for more information.

  For Table 4, we similarly preserved the original whole table as a new Supplementary Table 2, in the paper cut down Table 4 to only show the “series but not flu” outcome, and refer readers to Supplementary Table 2 for more information.

I find the predicted probabilities much easier to interpret than the the beta coefficients, and suggest focusing on those results. The figures are particuarly helpful.

- In Table 3, the beta coefficients actually represent change in probabilities (e.g., “0.116” means probability increase of 0.116 / 11.6% absolute increase). We changed the column title from “β” to “ΔPr.” to make this more clear, and mention this plus an interpretation example in the footnote.

Discussion:

The strengths and limitations are adequately discussed.

- Thank you!

**Comments from Reviewer #2**:

Reviewer #2: This is a generally well written manuscript describing what appears to be a very well designed and conducted study (although I am unable to comment definitively on the appropriateness/rigour of the statistical analyses as this is not my particular area of expertise) exploring an important area where previously published research is limited, and with findings that should be of broad interest. A few suggestions to enhance the paper are provided.

- Thank you for the accurate summary and kind words of the importance and quality of the work.
- We have addressed your section-by-section comments below.

Introduction

The sentence “Further, “complete uptake” – receiving the appropriate number of influenza vaccinations for the child’s age – is much lower, peaking at 45% in children 6-23 months old” is confusing. Children aged <9 years require 2 doses in their first season of vaccination so the number of doses of influenza vaccination recommended in any particular season depends on both the age and number of doses previously received. The paper cited looked at children aged 6-23 months from 2002 to 2012 and found that complete vaccination in this age group peaked at 45% in 2011/12, which is a quite different conclusion from that noted above. Complete vaccination for children aged 2-<5 years, who will often only need one dose, will be higher. Suggest reword this sentence.

- Thank you. We revised the sentence to be more clear and accurate in a general sense:

Further, “complete uptake” as defined by the CDC – receiving the appropriate number of influenza vaccinations for the child’s age and birthdate – is generally much lower in young children.[24]

- Regardless, the general comparison made at the end of the introduction across the two paragraphs as summarized below still accurately highlights the problem:

  “That the 19-shot, 7-vaccine series (4:3:1:3:3:1:4) uptake rate is comparable to the recent 2018/2019 single-season peak in influenza vaccine uptake in young children indicates unique mechanisms affect parents’ decisions to vaccinate their child against influenza relative to every other routinely-recommended childhood vaccine…”

Methods

It is not clear how ‘complete influenza vaccination’ has been assessed. This is described as “whether the child received the full number of influenza vaccines given the number of influenza seasons they have experienced by their second birthday”. However as noted above this depends on both age and number of doses previously received. In this age group, if assessing ‘complete influenza vaccination’ for the most recent influenza season this would require two doses to have been received unless two doses had been received in the previous season, in which case would only require one dose. Suggest reword to make clearer how assessed.

- Thanks for asking for clarification. The influenza vaccination outcome variable is defined by the US federal government to assess complete child influenza vaccination coverage by the child’s second birthday. It is based off of the child’s birthdate, and whether they have had the appropriate number of seasonal flu vaccine by their second birthday across however many flu seasons that two-year period covered and depending on when the survey questions was asked to them (i.e., the number of vaccines required to be considered “up to date” can vary depending on birthdate and survey administration date). Nonetheless, it is vetted by the CDC. The full description is below from the CDC survey manual ([click here and see pages 65-66](ftp://ftp.cdc.gov/pub/health_statistics/nchs/dataset_documentation/nis/nispuf11_dug.pdf), also source 38 in the paper):

“Vaccinated – For interviews conducted during year x (defined using year variable associated with the quarter), child was of age between 6 and 23 months during the entire span from 9/1 through 12/31 of year x-1, and either a) received no doses of influenza vaccine prior to 9/1/x-1, but then received two between 9/1/(x-1) and whichever is earlier, date of interview or 1/31/x or b) received at least one dose of influenza vaccine prior to 9/1/x-1 and then received one during the period 9/1/x-1 through 12/31/x-1.

Not vaccinated – For interviews conducted during year x (defined using year variable associated with the quarter), child was of age between 6 and 23 months during the entire span from 9/1 through 12/31 of year x-1, but does not qualify for the above definition.

Not eligible – For interviews conducted during year x (defined using year variable associated with the quarter), child's age fell outside the span of 6 and 23 months at any point between 9/1/x-1 and 12/31/x-1.”

- For the reader’s aid, we clarified and added the following guided language to the “Dependent variable” sub-section of the Methods section, including where to find more information:

  “The first is complete influenza vaccination – that is, whether the child received the full number of **seasonal** influenza vaccines given the number of influenza seasons they have experienced by their second birthday **and when the survey was administered** (children not 6-23 months of age during the span of September 1 to December 31 are “not eligible;” **see Section 7.8.1 and Table 7 of the survey user’s guide [38]**).”

Discussion

2nd para states that the unique determinant of hidden vulnerability to flu identified (parental history of never delaying vaccination) “may reflect the rise in vaccine hesitancy”. However, influenza vaccination rates in children have always been lower than other vaccines, likely reflecting longstanding hesitancy specific to this vaccine. Suggest delete this wording – the remainder of the relevant sentence stands alone without this somewhat dubious assertion.

- Thank you for pointing out this nuance, which we have reflected in the revisions below:
- ~~This may reflect the rise in~~ While vaccine hesitancy **has risen recently**,[65] **child influenza vaccination rates have been lower than other vaccines for quite some time and our finding was independent of general vaccine hesitancy**. **This finding likely represents longstanding hesitancy specific to the influenza vaccine.** ~~in a way not observed before~~ **P**erhaps many parents with children UTD on most vaccines, who thus appear to support the concept of vaccination, are uniquely hesitant or skeptical about the influenza vaccine.

There is much mention of parental attitudes to vaccination however no mention of potential impact of provider attitudes to influenza vaccination. I am unaware what research has been conducted in this area in the US, but there is certainly evidence from other countries that ‘hesitancy’ amongst providers regarding influenza vaccination is higher than for other vaccines, particularly related to the comparatively low effectiveness of influenza vaccine, which may impact on recommendation/ strength of recommendation. This is important given the extensive evidence that strong provider recommendation is a key determinant of vaccine uptake. While parent perception of strength of physician’s vaccine recommendation was included as a variable in this study, with no significant associations detected, I assume that this was framed in the survey in general terms rather than specific to particular vaccines such as influenza. Suggest add brief discussion of this issue to the limitations section.

- You correctly point out that we do control for parental perception of the strength of physician vaccine recommendation and find it not associated with any up-to-date status outcomes, but that the questions are about vaccination generally and not specific to the influenza vaccine.
- We now add this as a limitation (including that it could explain the lack of findings relative to the physician strength variables) and thank you for finding this important gap in our limitations assessment. See the following addition to the limitations section:

  “Fourth, the parental concerns variables refer to vaccination generally and not to any one specific vaccine, which could explain some of the non-findings (such as parent perception of physician recommendation for vaccination not being associated with our outcomes, contradicting other studies [27,41,45–50,52–56]).

General comment – suggest a thorough proof read as there are a moderate number of typographical and grammatical errors.

- We performed additional proofreading and corrected of typographical and grammatical errors.
